# Self-emulsifying Drug Delivery System for Improved Dissolution and Oral Absorption of Quetiapine Fumarate: Investigation of Drug Release Mechanism and* In-vitro *Intestinal Permeability

**DOI:** 10.22037/ijpr.2021.114785.15032

**Published:** 2021

**Authors:** Olfa Ben Hadj Ayed, Mohamed Ali Lassoued, Badr Bahloul, Souad Sfar

**Affiliations:** *Laboratory of Pharmaceutical, Chemical and Pharmacological Drug Development LR12ES09, Faculty of Pharmacy, University of Monastir, Avicenne Street, 5000 Monastir, Tunisia.*

**Keywords:** Quetiapine fumarate, self-emulsifying drug delivery system, release kinetics, D-optimal mixture design, Everted Gut Sac

## Abstract

In this study, we focused on quetiapine fumarate (QTF), a class II BCS drug. QTF is an atypical antipsychotic used in the treatment of schizophrenia and bipolar disorders. Our objective was to develop a new QTF-loaded self-emulsifying drug delivery system (SEDDS) to improve the dissolution and absorption of the drug. An experimental design approach was used to develop and optimize QTF-loaded SEDDS. The optimized formulation was characterized for droplets size, zeta potential, PDI, and stability. It was then evaluated using an *in-vitro *combined test for dissolution and Everted gut sac technique. Mathematical modeling and Transmission electron microscopy (TEM) were used to elucidate the mechanism of release.

The optimal formulation was type IIIB SEDDS, constituted of 9.1% of oleic acid, 51.6% of Tween^®^20, and 39.3% of Transcutol^®^ P. It showed a droplets size of 144.8 ± 4.9nm with an acceptable PDI and zeta potential. For *in-vitro *evaluation tests, we noticed an enhancement of the dissolution rate of the optimal QTF-loaded SEDDS compared to the free drug (98.82 ± 1.24% for SEDDS after 30 min compared to 85.65 ± 2.5% for the pure drug). The release of QTF fitted with the Hopfenberg model indicating the drug was released by water diffusion and erosion mechanism. This result was confirmed by TEM images which showed a smaller droplet size after release. We also found an amelioration of the permeability of QTF of 1.69-fold from SEDDS compared to the free drug. Hence, the SEDDS formulation represented a new way to improve the dissolution and absorption of QTF.

## Introduction

Psychotic disorders like schizophrenia are defined as severe and chronic mental disorders where the patient loses his capacity to behave, think, and feel normal. These diseases deeply affect the daily life of patients, their relations, and their well-being. All these factors explain the importance to treat these affections (1, 2).

Quetiapine, commercialized as a fumarate salt (QTF), is one of the recent molecules used principally for the treatment of schizophrenia and bipolar disorders. QTF is a dibenzothiazepine derivative belonging to the family of atypical antipsychotics. It was approved by the Food and Drug Administration (FDA) in 1997, and it showed good efficacy and better tolerability than classical antipsychotics such as chlorpromazine and haloperidol (3, 4).

Quetiapine and its metabolite N-desalkyl Quetiapine have a clozapine-like activity; they are antagonists of many neurotransmitter receptors, mainly serotoninergic receptors 5HT_2 _and dopaminergic receptors D_1_ and D_2_. This antagonism is the main responsible factor of the antipsychotic effect. Additionally, Quetiapine has a low affinity to α-adrenergic and H1 histaminergic receptors and practically no affinity to cholinergic muscarinic receptors. These properties allowed reducing the side effects of the drug-like cholinergic effects (5). All these properties make QTF an interesting molecule for the treatment of these chronic diseases.

QTF belongs to class II of the biopharm-aceutical classification system (BCS). It is slightly soluble in water. After oral administration, QTF is well absorbed and has a mean half-life of 6 hours. The major part of the absorbed fraction is metabolized in the liver principally by cytochrome (CYP) P450 3A4 (3, 6), and less than 5% of QTF are excreted in urine as an unchanged drug. This important hepatic metabolism in addition to the poor solubility, resulted in a poor oral bioavailability (9%) of the drug (7, 8). 

To overcome this problem, many technologies have been employed to develop diversified formulations that bypass the first hepatic passage and improve the oral bioavailability of QTF (7-9). Among these formulations, self-emulsifying drug delivery systems (SEDDS) are a new promising type of formulations that have known a big interest in the last years (10). SEDDS are defined as lipid-based systems composed of a mixture of oil and surfactants, and optionally cosurfactants and cosolvents, that in contact with an aqueous phase like digestive liquid, and under gentle agitation simulating the gastrointestinal tract movements, will form a fine and stable emulsion (11, 12). Among many advantages, SEDDS has shown a good ability to improve intestinal absorption of diverse drugs (13). The role of oil-in-water (O/W) emulsions in improving the intestinal absorption of drugs have been proven in many studies (10, 14, 15), but the instability of this form was the major factor limiting its use. With the apparition of self-emulsifying systems, the problem of the stability of these formulations has been solved since the emulsion is formed only before administration (16). SEDDS are also known to improve the intestinal lymphatic passage of many molecules and hence, to avoid the first hepatic metabolism (17-19). Moreover, it has been reported that SEDDS are capable of improving the solubility of poorly soluble molecules. Different mechanisms could explain this important ability of SEDDS in enhancing the solubilization of drugs.

In this study, we aimed to develop and optimize a new SEDDS formulation of QTF using a quality-by-design approach. We also explored the drug release mechanism from the optimized SEDDS formulation, and we evaluated the *in-vitro *intestinal permeability using the rat everted gut sac technique

## Experimental

*Reagents*

QTF was a gift from “Philadelphia Pharma” laboratories (Sfax, Tunisia); purified oleic acid and Tween^®^ 20 (polysorbate 20) were purchased from Prolabo^®^ (Paris, France); Transcutol^®^ P (diethylene glycol monoethyl ether) was provided by Gattefosse^®^ (Saint-Priest, France). All other chemicals used were of analytical grade.

*Formulation and optimization of QTF-loaded SEDDS*

*Construction of ternary phase diagram*

A ternary phase diagram was constructed to delimit the concentration intervals of components that define the self-emulsifying region. The components of the formulation were selected based on their ability to solubilize QTF. Thus, oleic acid, Tween^®^ 20, and Transcutol^®^ P were used as an oil, surfactant, and cosolvent, respectively. 

*Oily phase preparation*

 A series of unloaded SEDDS formulations were prepared by varying the percentage of each component in the preparation and keeping a final sum of concentrations of 100%. The intervals of work for oleic acid, Tween^®^ 20, and Transcutol^®^ P were respectively 5-70%, 20-70%, and 10-75% (m/m). First, oleic acid was introduced into a test tube, then the cosolvent and the surfactant were added successively under vortexing. The mixtures were vortexed for 2 minutes to obtain clear homogenized preparations and were let to stabilize at room temperature.

*Self-emulsifying capacity*

All the prepared formulations were evaluated for self-emulsifying capacity according to Craig et al. method (20). Briefly, 50 µL of each mixture was introduced into 50 mL of distilled water preheated at 37 ± 0.5 °C. The preparation was gently stirred at 100 rpm for 5 min using a magnetic hot plate stirrer (IKA^®^ RH Basic 2). Every preparation was then classified based on its tendency to spontaneous emulsification and its stability. Three grades of self-emulsifying capacity were predefined (Table 1). The preparations with “good” or “moderate” self-emulsifying capacity were then assessed for droplet size measurement. Only preparations with droplet sizes ranged between 100 and 300 nm were accepted for further studies.

*Drug incorporation*

QTF loaded-SEDDS were prepared by adding 20 mg of QTF to 1 g of the unloaded formulation. First, QTF was added to the amount of Transcutol^®^ P and stirred using a magnetic stirrer (IKA^®^ RH Basic 2) for 5 min at 50 °C. Then, oleic acid and Tween^®^ 20 were added to the mixture, respectively. The preparation was maintained under stirring for 20 min until the total solubilization of the drug.

The loaded preparations were then evaluated for self-emulsifying capacity, droplet size, and polydispersity index (PDI). Only formulations with droplets size between 100 and 300 nm were accepted for later optimization.

*Droplet size measurement*

Droplet size and PDI were measured by the dynamic light scattering method using a Nanosizer^®^ instrument (Nano S, Malvern Instruments, UK). The preparations were measured directly after reconstitution. All measurements were repeated three times (n = 3). Results were expressed as mean ± SD.

*Optimization of QTF-loaded SEDDS using D-optimal mixture design*

To optimize the SEDDS composition, a D-optimal mixture design was employed. This design was selected for its property to variate the proportion of each factor without changing the total sum of components (100%). In our case, the percentages of each component were defined as the independent variables of the design: oleic acid (oil% w/w; X1), Tween^®^ 20 (surfactant%w/w; X2), and Transcutol^®^ P (cosolvent% w/w; X3). The low and high levels of each independent variable were fixed based on the ternary phase diagram results. Mean droplets size (Y1) and PDI (Y2) were selected as responses to evaluate and optimize SEDDS characteristics. The Design Expert^®^ (Version 10, Trial version, Stat-Ease Inc., Minneapolis, MN, USA) software was employed for the statistical analysis. The software generated sixteen experimental runs that were prepared as described previously and assessed for both responses Y1 and Y2.

The polynomial equations of each response were provided by Design Expert^®^ software after data processing using analysis of variance (ANOVA). The selection of the best fitting mathematical models was based on the comparison of several statistical parameters such as sequential *p-value*, lack of fit *p-value*, squared correlation coefficient (R^2^), adjusted R^2^, predicted R^2^, and the predicted residual sum of square (PRESS). PRESS indicates how well the model fits the data. The selected model must have the smallest PRESS value compared to the other models under consideration (21, 22). Finally, the optimization of the three independent variables for both responses was accomplished by using the desirability function of the Design Expert^®^ software.

*Optimal QTF-loaded SEDDS characterization*

The optimal QTF-loaded formulation was prepared and reconstituted as described above. The reconstituted formulation was characterized for droplet size, PDI, zeta potential, and percentage of transmittance.

*Droplet size and PDI measurement*

Droplets size determination was assessed using the dynamic light scattering method using a Nanosizer^®^ (Nano S, Malvern Instruments, UK). Results were expressed as mean ± SD of three repetitions (n = 3).

*Zeta potential measurement*

The zeta potential value was determined by the dynamic light scattering technique using a Zetasizer^®^ (Nano Z, Malvern Instruments, UK). The measurements were run in triplicate, and results were expressed as mean ± SD.

*Transmittance*

The transmittance percentage was measured using a UV-Visible spectrophotometer (Evolution 60, Thermo Scientific) at λ = 650 nm to evaluate the transparency of the optimal SEDDS formulation. Purified water was used as the reference. Results were expressed as mean ± SD of three measurements (n = 3).

*Stability study*

To assess the stability of the optimal SEDDS formulation, three different assays were performed on both oily and reconstituted preparations. The formulations were evaluated under accelerated conditions such as centrifugation and freeze-thaw cycles and under normal storage conditions for one month.

*Stability to centrifugation*

 One and half milliliters of the oily phase or the reconstituted preparation were introduced into an Eppendorf tube and centrifuged at 10000 rpm for 15 min. The preparations were then inspected visually for the presence of precipitate of the drug, phase separation, or other visual instabilities.

*Stability to Freeze-Thaw cycles*

 Four milliliters of the oily phase or the reconstituted preparation were introduced into a hemolysis tube. Samples were then subjected to 3 freeze-thaw cycles of 48 h each, alternating 24 h at -10 °C and 24 h at room temperature. The preparations were then examined visually.

*Stability under normal storage conditions*

 The optimal SEDDS oily preparation was stored at room temperature for 30 days. Then, it was reconstituted (50 μL in 50 mL of distilled water at 37 °C) and checked for droplet size, PDI, and zeta potential.

*Transmission electron microscopy (TEM)*

The morphology of the oily droplets of the reconstituted optimal formulation was investigated by transmission electron microscopy. The SEDDS formulation was diluted 1000 times in preheated distilled water (37 °C) under magnetic stirring. After 15 min, a sample of 10 µL was withdrawn and placed on a copper-mesh grid and let to stand for 2 min. The excess was then removed by adsorbing on a filter paper. Ten microliters of 1% uranyl acetate solution were added to the grids for contrast and let to stand for 5 sec before removing the excess. The sample was observed using a JEM-1400 Transmission Electron Microscope (JEOL Ltd., USA).

For the QTF release mechanism study, the reconstituted formulation was kept under magnetic stirring (Ika^®^ RH basic 2 hot stirring plate, Germany) for 60 min at 37 °C. Then, another sample was withdrawn, prepared as described above, and observed under TEM for eventual morphologic modifications.

*Dissolution and permeation studies*

To study the release profile and the permeation behavior of QTF from the optimal SEDDS formulation, a combined dissolution, and permeation assay was designed and conducted using a rat Everted Gut Sac (EGS) permeability technique and USP dissolution apparatus I (Basket apparatus) method.

*Animals*

Male Wistar rats (200-250 g) aged between 8 and 12 weeks were used for the permeability study. Animals were purchased from the Central Pharmacy of Tunisia (Tunis, Tunisia) and were kept in standard environmental conditions in polypropylene cages at a controlled temperature (22-24 °C) with 12 h of light/dark cycles. They had free access to food and water. Before the experiment, the rats have fasted for 24 h with free access to water.

All experiments were performed according to the guidelines of the European Union on Animal Care (CCE Council 86/609).

*In-vitro dissolution and permeation studies using rat Everted Gut Sac model*

The EGS technique was conducted according to the method of Lassoued *et al.* (23, 24). Before the experiment, the fasted rats were anesthetized using ether. Then, a 3 cm incision was made in the abdomen of the rat. The jejunum was located, separated from the rest of the intestine, and cut into segments of approximately 6 cm in length. After the extraction of the intestine, the rat was immediately euthanized by over-exposure to ether. The intestine segments were rapidly incubated in an oxygenated (O_2_/CO_2_, 95%: 5%) Tyrode buffer solution (containing in mM: 15 glucose, 11.90 HCO_3_Na, 136.9 NaCl, 4.2 NaH_2_PO_4_, 2.7 KCl, 1.2 CaCl_2_ and 0.5 MgCl_2_) at 37 ± 0.5 °C. The sacs were washed three times with Tyrode solution, stripped of adhering tissues, and carefully everted over a thin cannula. One extremity of each sac was ligated with a silk thread, and the other extremity was tied to a small cannula allowing to fill the sac with Tyrode solution.

**Figure 1:**
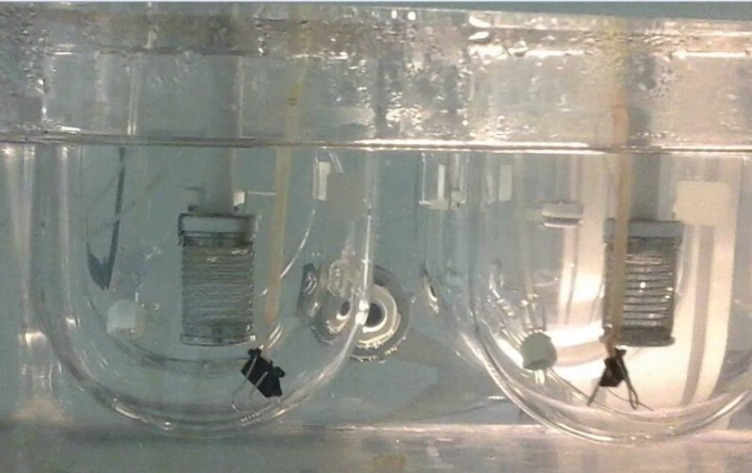
The system used for dissolution and permeation studies showing rat everted gut sac hanged into dissolution apparatus type II in vertical position containing Tyrode solution. The medium is constantly oxygenated through perfusion tubes

Each everted sac was filled with 500 µL of Tyrode buffer solution (Receiver compartment; pH 7.4) using a 1 mL syringe, and carefully hung into the dissolution apparatus recipient (basket apparatus ERWEKA GmbH, Heusenstamm, Germany) containing 900 mL of distilled water preheated at 37 ± 0.5 °C and oxygenated using perfusion tubes (O_2_/CO_2_, 95%: 5%). Small clumps were attached to the free end of the sacs to keep them submerged in the liquid in a vertical position (Figure 1). The optimal SEDDS formulation or the free QTF, equivalent to 50 mg of Quetiapine free base, were then added to the dissolution medium (Donor compartment) and stirred at 100 rpm.

At regular time intervals (10, 20,30,40,50, and 60 min), 3 mL aliquots were withdrawn from the donor medium and filtrated through a 0.1 µm nitrocellulose membrane. Simultaneously, an intestinal sac was removed, and its content was collected into an Eppendorf tube and centrifuged at 14 000 rpm for 10 min. The amount of drug in each sample was analyzed after suitable dilution, using a UV-Visible spectrophotometer (Evolution 60, Thermo Fisher Scientific) at 220 nm.

Results were expressed as mean ± SD of 6 repetitions (n = 6) for the *in-vitro *dissolution assay and as mean ± SD of 3 repetitions (n = 3) for the permeability assay.

*Apparent permeability calculation (P*_app_ *)*

The apparent permeability coefficient (P_app_) was calculated as follows (23, 25) :

$P_{\mathrm{app}}=\frac{\mathrm{dQ}}{\mathrm{dt}}\times \frac{1}{AC_{0}}$

Where P_app_ (cm/s) is the apparent permeability coefficient, dQ/dt (μg/s) is the amount of drug absorbed by unit of time, A (cm^2^) is the surface area available for permeation, and C_0_ (μg/mL) is the initial concentration of QTF in the donor compartment.

*Dissolution and diffusion profiles study*

The dissolution and diffusion profiles of both free drug and optimal formulation were compared using the model-independent mathematical approach using difference factor (*f*_1_) and similarity factor (*f*_2_*)*, proposed by Moore and Flanner (1996) (26):

f1=∑t=1nRt-Tt∑t=1nRt×100

f2=50×log 1+1n∑t=1nRt-Tt2-0.5×100

Where *R*_t_ and *T*_t_ are the percentages of drug released or diffused of the reference or the test formulation, respectively, at time t; and n is the number of time points.

The difference factor (*f*_1_) calculates the percentage of the difference between the two curves at each time point. It is a measurement of relative error between both curves. The similarity factor (*f*_2_) is a logarithmic reciprocal square root transformation of the sum of squared error. It represents a measurement of the similarity in the released percentage between the two curves. Two curves were considered similar when the *f*_1_ value was less than 15%, and the *f*_2_ value was greater than 50% curves.

*Mathematical Modeling of drug release kinetics*

The *in-vitro *dissolution data of optimal formulation was fitted to various release kinetic models (zero-order, first-order, Higuchi, Korsmeyer-Peppas, Weibull, and Hopfenberg models) to provide an insight on the drug release mechanism. The model-fitting analysis was accomplished using DDsolver^®^, a Microsoft^®^ Excel^®^ add-in program to model and compare drug dissolution profiles. The following equations were used for the explored models:

${\mathrm{Zero}-\mathrm{order}:Q}_{t}=Q_{0}+\mathrm{k.t}$

$\mathrm{First Order}:\log Q_{t}=\log Q_{0}+\frac{\mathrm{k.t}}{2.303}$

$\mathrm{Korsmeyer}-\mathrm{Peppas}:\frac{Q_{t}}{Q_{\infty }}=\mathrm{k.}t^{n}$

$\mathrm{Weibull}:Q_{t}=Q_{0}(1-e^{-\left(\frac{T}{T_{d}}\right)\beta })$

$\mathrm{Hopfenberg}:\frac{Q_{t}}{Q_{\infty }}=1-({1-\frac{\mathrm{k.t}}{C_{0}.a_{0}})}^{n}$

Where is the amount of drug dissolved in time t, is the initial amount of drug in the solution, is the fraction of the drug released at time t, *k* is the release rate constant, n is the release exponent, is the time required to dissolve 63,2% of the drug, β is the shape parameter, *C*_0_ is the initial concentration of the drug, *a*_0_ is the initial radio of a sphere or a cylinder or half-thickness of a slab, and *n* has a value of 1, 2 and 3 for a slab, cylinder and sphere, respectively.

The adjusted coefficient of determination (R^2^_adj_) was used to assess the fit of the models’ equations (27). It is calculated using the followed equation:

$R_{\mathrm{adj}}^{2}=1-\frac{\left(n-1\right)}{\left(n-p\right)}(1-R^{2})$

Where *n *is the number of dissolution data points *p *is the number of parameters in the model.

The best model is the one with the highest R^2^_adj_ value. The Akaike’s information criterion (AIC) described by the equation below was also examined to ensure the model’s suitability. The smaller the AIC, the better the model adjusts the data.

$\mathrm{AIC}=\mathrm{n.}\ln\left(\mathrm{WSS}\right)+\mathrm{2.p}$

Where n is the number of data points, WSS is the weighted sum of squares, and p is the number of parameters in the model.

*Statistical analysis*

Statistical analysis of the dissolution and the permeability studies was conducted using Microsoft Excel 2010 software. The Student’s t-test was used to evaluate the significant differences. A significant difference was considered when the *p-value* was ≤ 0.05.

## Results and Discussion

*Formulation and optimization of QTF loaded-SEDDS*

*Ternary phase diagram construction*

Oleic acid, Tween^®^ 20, and Transcutol^®^ P were selected as oil, surfactant, and cosolvent, respectively. The choice of excipients was based on their ability to solubilize QTF and their miscibility, tolerability, and safety towards the human body (7, 28 and 29). Oleic acid is a long-chain fatty acid that was largely used in lipid-based formulations for its capacity to improve oral bioavailability and enhance the intestinal absorption of drugs (30, 31). Oleic acid also has a good solubilization capacity of QTF, as reported in previous studies (8, 32). Tween^®^ 20 was selected as a surfactant in the formulation based on preliminary studies (data not shown). Tween 20 is a non-ionic surfactant with a high hydrophilic-lipophilic balance (HLB) value of 16.7. surfactants with high HLB values are known to facilitate the formation of small droplet size O/W emulsions and facilitate the spreadability of SEDDS formulations (33). Moreover, The non-ionic character of Tween^®^ 20 makes it less harmful to the intestinal barrier than other ionic surfactants (10). Transcutol^®^ P is a permeability enhancer and is known to be a very good and safe solubilizer of many drugs. Both Tween 20 and Transcutol^®^ P have shown a good solubilizing capacity of QTF (32).

**Figure 2:**
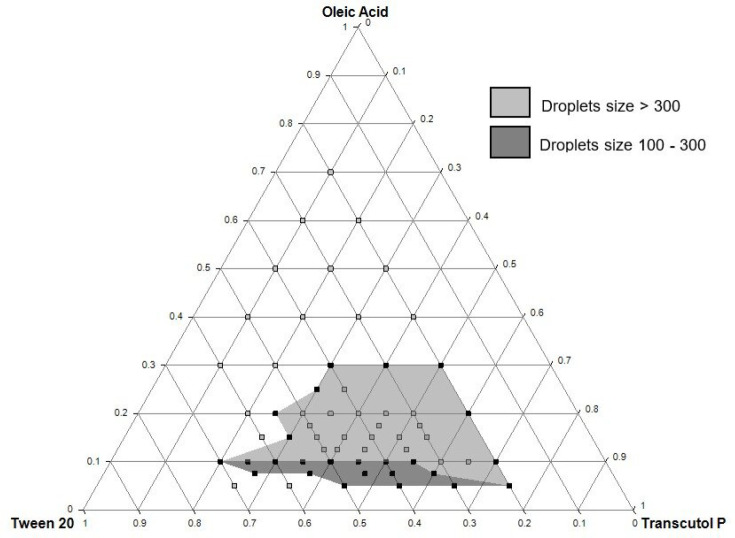
Ternary phase diagram composed of Oleic acid (oil), Tween 20 (surfactant), and Transcutol P (cosolvent). Both light grey (droplets size > 300 nm) and dark grey (droplets size between 100 and 300 nm) represent the self-emulsifying region

The ternary phase diagram was constructed to determine the self-emulsifying zone using unloaded formulations. As shown in Figure 2, the self-emulsifying zone was obtained within the intervals of 5 to 30% of oleic acid, 20 to 70% of Tween^®^ 20, and 20 to 75% of Transcutol^®^ P. The grey colored zone in the diagram shows the formulations that gave a “good” or “moderate” self-emulsifying capacity as reported in Table 1. The dark grey zone was delimited after drug incorporation and droplet size measurements and represented the QTF-loaded formulations with a droplet size ranged between 100 and 300 nm. These results served as a preliminary study for further optimization of SEDDS using the experimental design approach.

*D-optimal mixture design: statistical analysis*

D-optimal mixture design was chosen to optimize the formulation of QTF-loaded SEDDS. This experimental design represents an efficient technique of surface response methodology. It is employed to study the effect of the formulation components on the characteristics of the prepared SEDDS (34, 35). In D-optimal algorithms, the determinate information matrix is maximized, and the generalized variance is minimized. The optimality of the design allows making the adjustments required to the experiment since the difference of high and low levels are not the same for all the mixture components (36).

The percentages of the three components of SEDDS formulation were used as the independent variables and are presented in Table 2. The low and high levels of each variable were: 6.5 to 10% for oleic acid, 34 to 70 % for Tween^®^ 20, and 20 to 59.5 % for Transcutol^®^ P. Droplet size and PDI were defined as responses Y_1_ and Y_2_, respectively.

The Design-Expert® software provided 16 experiments. Each experiment was prepared and tested for droplet size and PDI. As shown in Table 3, values were comprised between 18.2 and 352.7 nm for droplet size and between 0.172 and 0.592 for PDI.

Droplet size and PDI results of each experiment were introduced and analyzed using the experimental design software. Both responses were fitted to linear, quadratic, special cubic, and cubic models using the Design-Expert^®^ software. The results of the statistical analyses are reported in the supplementary data Table S1. It can be observed that the special cubic model presented the smallest PRESS value for both droplet size and PDI responses. In addition, the sequential p-values of each response were < 0.0001, which means that the model terms were significant. Also, the lack of fit *p-values* (0.0794 for droplet size and 0.6533 for PDI) were both not significant (>0.05). The R² values were 0.957 and 0.947 for Y_1_ and Y_2_, respectively. The differences between the Predicted-R² and the Adjusted-R² were less than 0.2, indicating a good model fit. The adequate precision values were both greater than 4 (19.790 and 15.083 for droplet size and PDI, respectively), indicating an acceptable signal-to-noise ratio. These results confirm the adequacy of the use of the special cubic model for both responses. Hence, it was adopted for the determination of polynomial equations and further analyses.

*Influence of independent variables on droplet size and PDI*

The correlations between the coefficient values of X_1,_ X_2_, and X_3_ and the responses were established by ANOVA. The *p-values *of the different factors are reported in Table 4. As shown in the table, the interactions with a *p-value* of less than 0.05 significantly affect the response, indicating synergy between the independent factors.

The polynomial equations of each response fitted using ANOVA were as follows:

Droplet size: Y_1_ = 4069,19 X_1_ – 100,97 X_2_ + 153,22 X_3_ – 1326,92 X_1_X_2_ – 2200,88 X_1_X_3_ + 335,62 X_2_X_3_ – 8271,76 X_1_X_2_X_3_                      (1)

PDI: Y_2 _= 38,79 X_1 _+ 0,019 X_2_ + 0,32 X_3 _– 37,13 X_1_X_3 _+ 1,54 X_2_X_3 _– 31,31 X_1_X_2_X_3_                     (2)

It can be observed from Equations 1 and 2 that the independent variable X_1_ has a positive effect on both droplet size and PDI. The magnitude of the X_1_ coefficient was the most pronounced of the three variables. This means that the droplet size increases when the percentage of oil in the formulation is increased. This can be explained by the creation of hydrophobic interactions between oily droplets when increasing the amount of oil (25). It can also be due to the nature of the lipid vehicle. It is known that the lipid chain length and the oil nature have an important impact on the emulsification properties and the size of the emulsion droplets. For example, mixed glycerides containing medium or long carbon chains have a better performance in SEDDS formulation than triglycerides. Also, free fatty acids present a better solvent capacity and dispersion properties than other triglycerides (10, 33). Medium-chain fatty acids are preferred over long-chain fatty acids mainly because of their good solubility and their better motility, which allows the obtention of larger self-emulsification regions (37, 38). In our study, we have chosen to work with oleic acid as the oily vehicle. Being a long-chain fatty acid, the use of oleic acid might result in the difficulty of the emulsification of SEDDS and explain the obtention of a small zone with good self-emulsification capacity.

On the other hand, the negativity and high magnitude of term coefficients containing the X_2_ factor suggested that the surfactant proportion had a negative effect on droplet size. Hence, an increase in Tween^®^ 20 concentration leads to a decrease in the size of oily droplets. Tween^®^ 20 is a high HLB value surfactant with a linear alkyl chain structure. Its short chain length (C12) and high hydrophilicity (HLB 16.7) provide more fluidity and flexibility to the interfacial film and hence, allow a greater ability to incorporate water and contribute to the rapid formation of oil droplets. These findings are in accordance with previous studies (25, 39). 

**Figure 3:**
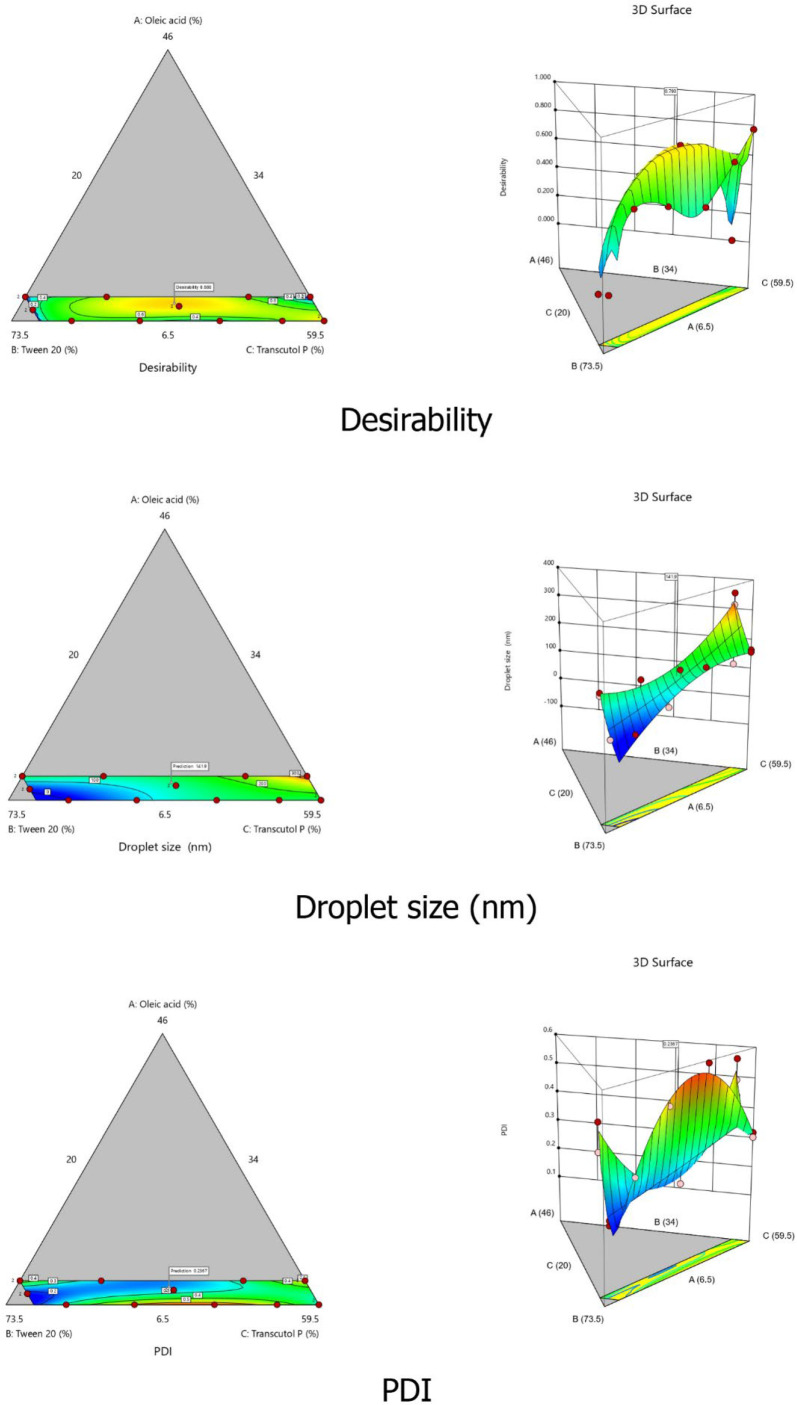
Contour plots (left) and 3D response surface plots (right) displaying the effect of independent factors on desirability, droplets size, and PDI

The combination of the three variables X1, X2, and X3 gave the maximum magnitude of coefficients, suggesting that the interaction between the components deeply affected the size of the droplets in the system (*p *< 0.05). These results can be confirmed by the 2D contour plots and the 3D graphical representations of both droplet size and PDI responses (Figure 3).

*Optimization of SEDDS formulation using desirability function*

The three independent variables X_1_, X_2_, and X_3_ were simultaneously optimized for both responses Y_1_ (droplets size) and Y_2 _(PDI) using the desirability function. The advantage of the desirability function is its ability to combine all responses in only one measure and allow predicting the optimum value of each variable based on the predefined criteria. In this work, we aimed to minimize the values of both responses within the predefined intervals of 100 nm to 300 nm for droplets size and less than 0.300 for PDI. We also opted to minimize the percentage of surfactant in the formulation to ensure the safety and tolerability of the formulation. Design Expert^®^ software provided three optimized formulations with reduced droplet size and reduced PDI values. The formulation that presented the smallest droplet size and the closest desirability value to 1 was retained as the optimal SEDDS formulation and used for further studies. The optimized percentages of the three independent variables X_1_, X_2_, and X_3_ were 9.07% (oil), 51.6% (surfactant), and 39.3% (cosolvent), respectively. The predicted droplet size and PDI values were 141.95 nm and 0.237, respectively, with a desirability value of 0.880 (Figure 3). To validate the predicted values of both responses, the optimal formulation was prepared and assessed for droplet size and PDI. The results of the correlation between the predicted and observed values were then analyzed using Student’s test. For droplet size, the predicted value was 141.95 nm compared to 144.8 ± 4.9 nm for the actual value with no significant difference (*p-*value = 0.077). The predicted PDI value was 0.237, and the actual value was 0.327 ± 0.046. Although the variation of PDI value was moderately high, the *p-*value (0.414 > 0.05) indicated a non-significant variation. Consequently, the chosen formulation was validated and adopted for further studies (Table S2).

*Characterization of the optimized QTF-loaded SEDDS*

Referring to the proposed classification system of Pouton for lipid-based formulations (40, 41), the selected optimal formulation can be defined as type IIIB formulation with an oil percentage less than 20%, a surfactant percentage approximatively ranged from 20 to 50%, and a cosolvent percentage ranged from 20 to 50%.

Table 5 summarizes the results of the characterization of the optimal QTF-loaded SEDDS.

The preparation presented a droplet size of 144.8 ± 4.9 nm and a PDI value of 0.327 ± 0.046. The small droplet size of the formulation confirms its suitability for oral delivery. The PDI was close to 0.3 and indicated homogenous distribution of the size of droplets (42).

The zeta potential value was -28.1 ± 0.32 mV indicating a negative charge of particles. The negativity of the charge in the surface of droplets could be explained by the presence of the polyoxyethylene group of the surfactant (43). In conventional emulsions, the zeta potential represents an important indicator of the stability of the preparation. It measures the electrical charge around the particles of emulsion, which represents the electric and electrostatic forces of repulsion and attraction between particles. High zeta potential values provoke electrostatic repulsive forces and prevent particles from flocculating, which contributes to the stability of the colloidal system (44). In our work, SEDDS presented a negative high value of zeta potential, indicating the stability of the developed system.

The developed formulation also presented a transmittance value of 97.7%, which indicates that the formulation has good transparency and consequently small droplets size (45).

**Figure 4:**
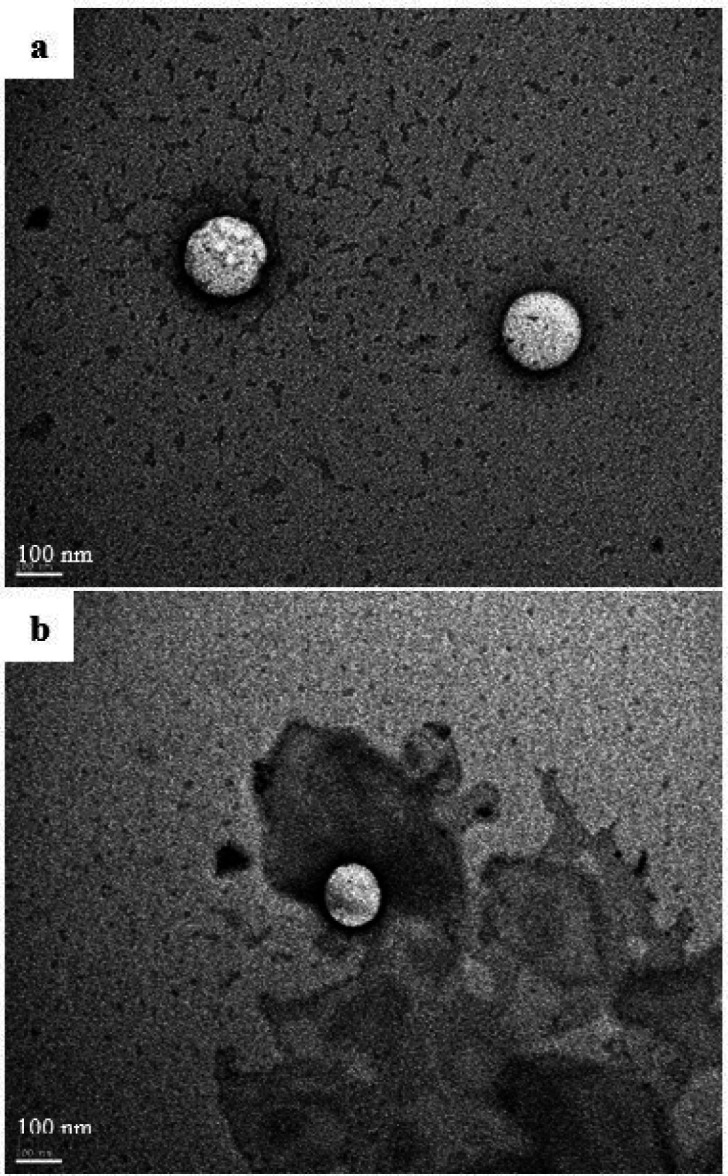
TEM images of the optimized formulation of QTF-Loaded SEDDS (a) after 15 min of reconstitution, magnification 100 000X; (b) after 60 minutes of the dissolution assay, magnification 100 000X

The morphological examination of the reconstituted self-emulsifying system by transmission electron microscopy is shown in Figure 4a. The images showed well-defined spherical droplets with a bright core referring to the oily phase. The dark shell surrounding the oil droplets represents the surfactant layer. The size of the droplets was homogenous and in good correlation with the Nanosizer^®^ measurements. 

*Stability study*

For the stability studies, both oily and reconstituted optimal preparations have shown good stability after three freeze-thaw cycles, without any phase separation or drug precipitation. Similarly, the centrifugation did not affect the visual aspect of the preparations. Hence, the formulation was considered stable. The accelerated stability tests are performed to anticipate the shelf-life of the formulation upon long-term storage at normal conditions (43). The centrifugation test stimulates the aging of the formulation using gravitational force, while the freeze-thaw cycles test accelerates the phase separation of the formulation by thermal treatment (46). The stability of the optimal formulation under these conditions allows predicting its stability upon storage for longer periods.

After one month of storage at room temperature, the formulation was reexamined. The oily preparation was stable and limpid. The reconstituted preparation represented a droplet size of 134.3 ± 6.3 nm with a PDI value of 0.395 ± 0.026 and a zeta potential of -27.8 ± 0.94 mV. The variations in droplet size, PDI, and zeta potential were not significant (*p-*value > 0.05), which proves the stability of the preparation.

The droplet size and zeta potential did not incur any significant changes compared to the first day of preparation, but a small elevation in PDI value was observed. In conclusion, at the normal storage conditions, the stability of the prepared SEDDS was not significantly affected.

*Dissolution and permeation study*

The EGS technique was widely employed in previous works by Lassoued *et al. *(23, 24). The experimental conditions (medium composition, temperature, and oxygenation) were optimized to guarantee the viability of the intestine during the assay. In this work, we have brought slight modifications to the method of Lassoued *et al.* (23) to optimize the technique and mimic a better physiological process of the formulation after oral administration (dissolution followed by absorption).

Thus, to evaluate the new formulation, dissolution and permeation tests were combined in one simultaneous test. This combination also allowed to reduce the number of experiments and consequently to minimize the variations due to experimental error.

*Dissolution study*

A dissolution study was conducted to compare the dissolution profile of the optimal SEDDS formulation with the free drug. The dissolution test was assessed in USP apparatus I. At different time intervals, samples were withdrawn for analysis. In the case of SEDDS, samples were pretreated by filtration (membrane filter porosity = 0.1 µm < oily droplet size) to separate the dissolved fraction of QTF from the fraction encapsulated in oily droplets.

**Figure 5:**
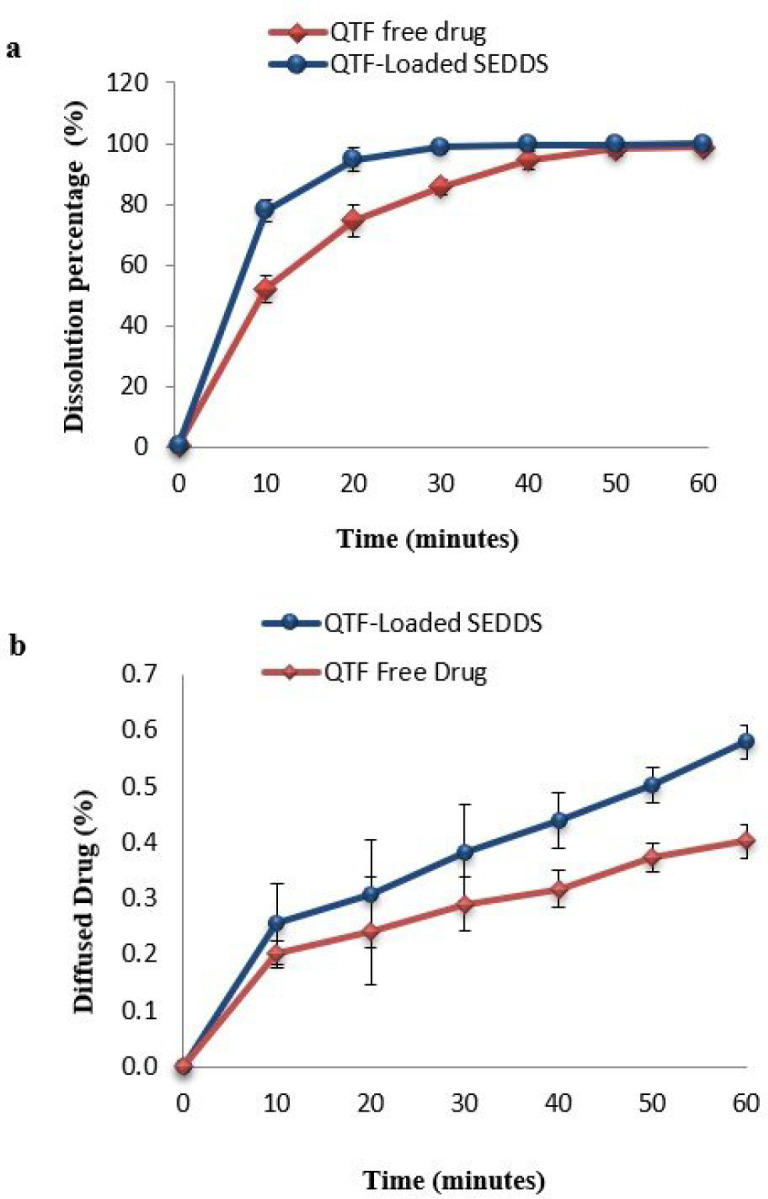
Dissolution and diffusion profiles of QTF free drug and optimal QTF loaded-SEDDS (a) Dissolution profile using type II dissolution apparatus in water (b) Diffusion profiles through rat everted gut sac membrane

The dissolution results showed an enhanced dissolution rate of SEDDS comparing to free QTF (Figure 5a). After 10 min, the dissolution of SEDDS (76.86 ± 3.61%) was remarkably higher than the dissolution of the free drug (52.23 ± 4.42%). The dissolution of SEDDS was almost complete after 30 minutes with a percentage of 98.82 ± 1.24%, while it was only 85.65 ± 2.5% for the free drug. After 60 min, the dissolution was complete for both forms. To compare the dissolution profiles of both free QTF and SEDDS, the similarity test was used. The calculated values of the difference factor (*f1) *and the similarity factor (*f*_2_) were 11.67% (*f*_1 _< 15%) and 43.54%* (f*_2_ < 50%), respectively, indicating the profiles were not similar. The role of SEDDS in enhancing the solubilization of poorly soluble drugs has been observed in several studies (25, 45). This could be explained by the presence of surfactant with high hydrophilicity (Tween^®^ 20), which facilitates the immediate formation of oily droplets in the aqueous medium after dispersion. In the presence of surfactant, solubilization and rapid water penetration within the oil phase will occur and lead to interface disruption and a decrease in the size of droplets (13, 47). This decrease provides a more important surface of exchange between oily droplets and aqueous medium and facilitates the dissolution of the drug (48).

*Mathematical Modeling of drug release kinetics*

To evaluate the release mechanism of QTF from optimal SEDDS formulation, the drug release data were fitted to various release kinetic models (zero-order, first-order, Higuchi, Korsmeyer-Peppas, Weibull, and Hopfenberg models). Table 6 summarizes the results of fitting data. The criterions used to select the appropriate model were R^2^_adj_ and AIC. The best-fitting model is the one with the highest R^2^_adj_ and the smallest AIC values. As shown in Table 6, the zero-order and Higuchi models did not give good data fitness with negative R^2^_adj_ values (-21.8729 and -5.3309 respectively) and high AIC values (55.9229 and 48.0458, respectively).

The best-fitting models were Weibull (R^2^_adj _= 0.9940) > Hopfenberg (R^2^_adj _= 0.9862) > first-order (R^2^_adj _= 0.9850), respectively. The AIC values are in good correlation with these results. The Weibull model had the smallest AIC value. The drug release profile fitted well with the first-order kinetics. This means that the amount of the drug released is proportional to the amount remaining in the oily droplets. Hence, it will diminish over time (27). This was shown by the dissolution profile where the drug follows a two-step release process, an initial burst release phase followed by a slower release phase (49).

For a better understanding of the release mechanism, the Weibull model was investigated. The β value is higher than 1 (1.41), indicating that a complex mechanism governs QTF release from the oily droplets. The T_d_ was 6.799, which means 63.2% of the drug was released from SEDDS in 6.799 min (50). These results were consistent with a previous study that investigated the release of gemfibrozil from SNEDDS formulation. The authors demonstrated that gemfibrozil release kinetics followed the Weibull model with a β value of 2.05 (51). Hence, the initial burst release phase could be attributed to the drug present at the surface of the oily droplets and entrapped in the surfactant layer, explained by the higher solubility of QTF in Tween 20 than in oleic acid (7).

The Hopfenberg model could support this theory, which also gave a good fitting of the release data. The Hopfenberg equation describes a heterogeneous erosion of the pharmaceutical form. Bahloul *et al.* (52) have studied the mechanism of release of fenofibrate from SEDDS formulation by investigating the structural changes in the shell and core of oil droplets using transmission electron microscopy. They suggested that, after dilution of SEDDS, the drug could be released by water diffusion and erosion mechanism by alteration of the arrangement of surfactant layer and ejection of smaller nanomaterial. These findings are in harmony with our mathematical modeling results and could explain the QTF release mechanism from the optimal SEDDS formulation. Moreover, the TEM analysis of the oil droplets of the reconstituted formulation after one hour of the dissolution assay showed a reduction in the size of droplets. This reduction could be explained by a loss of nanomaterial from the initial droplets (Figure 4b). These findings could confirm the suggested release mechanism.

*Permeability study*

For the permeability study, the EGS technique was performed to study the intestinal absorption of QTF. The EGS technique is an efficient method to evaluate the transport of drugs through the intestinal barrier (24). In our study, this technique was employed to investigate the intestinal absorption of QTF from the novel SEDDS formulation compared to the free drug. During the assay, the viability of the intestine segments was maintained by the use of Tyrode solution and continuous oxygenation. It was reported in previous studies that the intestine segments were maintained viable up to 90 min under these conditions (53, 54).

Figure 5b reports the diffusion profiles of both optimal formulation and free drug. The curves illustrate the percentage of the diffused drug through the intestine barrier over time during 60 min. The results showed a remarkable enhancing of the diffused drug in the case of SEDDS (0.579 ± 0.030%) compared to free QTF (0.402 ± 0.030%).

To compare the obtained profiles, a similarity test was established. The difference factor *f*_1_ and similarity factor *f*_2_ were 35.11% (*f*_1_ > 15%) and 99.86% (*f*_2_ > 50%), respectively, indicating that the curves were not similar, which confirms the significant difference between the two diffusion profiles (25). The calculation of P_app _coefficient has also demonstrated a significant improvement of 1.69-fold in the case of SEDDS (2.71 ± 0.47 10^-4^cm/s) compared to free QTF (1.6 ± 0.5 10^-4^cm/s) (*p* < 0.05). This enhancement could be attributed to the small size of the formed droplets since the reduction of the droplet size increase the surface of interaction with the intestinal barrier (55). Also, the use of Tween^®^ 20 as a surfactant could improve intestinal permeability by interfering with the lipid bilayer of the membrane of the epithelial cells. Surfactants act by changing the structural organization of the lipid bilayer of membranes, enhancing the fluidification of intestinal cell membranes, and opening the tight junctions (16, 56 and 57). The role of lipid drug delivery systems in enhancing QTF oral bioavailability has been studied previously, and similar results were found. Parvathi et al. developed a QTF oral microemulsion and found a 1.47-fold enhancement in the *in-vitro *release and the *ex-vivo* diffusion of the microemulsion compared to the drug suspension (58). Vadlamudi et al. also developed a QTF-based solidified self-microemulsifying system and demonstrated that the new formulation could improve the *in-vivo* antipsychotic activity of QTF in rats. They reported that this improvement could be attributed to the enhancement of the absorption of QTF from the new formulation compared to the free drug (59).

Moreover, the use of oleic acid as oil could have benefits on the improvement of the bioavailability of QTF. It is known that long-chain fatty acids like oleic acid are not directly transported into the blood circulation. After internalization into the enterocytes, these fatty acids are re-esterified to triglycerides, incorporated into chylomicrons, and then transported into the lymphatic system (17, 60). Hence, the associated drug molecules are transported into lymph vessels and bypass the hepatic first-pass metabolism, which contributes to the enhancement of the bioavailability of the drug (61, 62). 

**Table 1:** Visual evaluation of self-emulsification capacity (Craig *et al.* 1995) (20).

Self-emulsification capacity	Comments
Good	Spontaneous emulsification occurs immediately. Time of homogenization within 1 min. Formation of a transparent or almost transparent stable emulsion
Moderate	Spontaneous emulsification is less pronounced. Time of homogenization within 1 min. Formation of clear to stable white emulsion
Bad	Spontaneous emulsification does not occur; the oily phase forms a layer on the bottom or in the top. Time of homogenization over 3 min. Formation of a white milky instable emulsion

**Table 2:** D-optimal mixture design independent variables and identified levels

Independent variable	Excipient	Range (%)	
		Low level	High level
**X**_1_	Oleic Acid (%)	6,5	10
**X**_2_	Tween^®^20 (%)	34	70
**X**_3_	Transcutol^®^P (%)	20	59,5
	**Total**	100 %	

**Table 3:** Experimental matrix of D-optimal mixture design and observed responses

	Component 1	Component 2	Component 3	Response 1	Response 2
Experience number	**A: Oleic Acid%**	**B: Tween** ^®^ **20%**	**C: Transcutol** ^®^ **P%**	**Particle size (nm)**	**PDI**
**1**	10	34	56	352.73	0.559
**2**	8.64004	51.261	40.099	160.9	0.282
**3**	6.5	57.2885	36.2115	66.97	0.492
**4**	6.5	34	59.5	154.8	0.317
**5**	10	70	20	154.56	0.489
**6**	8.11183	70	21.8882	18.87	0.172
**7**	10	41.801	48.199	189.73	0.305
**8**	10	70	20	164.36	0.397
**9**	6.5	39.2781	54.2219	135.46	0.461
**10**	8.64004	51.261	40.099	132.2	0.216
**11**	6.5	65.9117	27.5883	18.2	0.307
**12**	6.5	34	59.5	163.2	0.301
**13**	10	34	56	312.76	0.489
**14**	6.5	47.1868	46.3132	155.83	0.592
**15**	8.11183	70	21.8882	18.49	0.188
**16**	10	59.7325	30.2675	161.96	0.301

**Table 4:** Summary of ANOVA for the special cubic model of the measured responses

Coefficient	Y_1_	*p-*value	Y_2_	*p-*value
**Linear mixture**	1.117E+05	<0.0001	0.0314	0.0048
**X**_1_ **X**_2_	11.06	0.8956	0.0087	0.0417
**X**_1_ **X**_3_	31.77	0.8242	0.0104	0.0290
**X**_2_ **X**_3_	4313.37	0.0258	0.0910	<0.0001
**X**_1_ **X**_2_ **X**_3_	7853.67	0.0058	0.1125	<0.0001

**Table 5:** Results of characterization of optimized QTF-loaded SEDDS

Parameters	Results	Commentary
%Transmittance	97.7%	
Droplet size (nm)	144.8 ± 4.9	
PDI	0.327 ± 0.046	
Zeta potential (mV)	-28.1 ± 0.32	
Stability to centrifugation	stable	Absence of precipitation or phase separation
Stability to Freeze-thaw cycles	stable	Absence of precipitation or phase separation
Stability at normal storage conditions	Droplet size = 134.3 ± 6.3 nm; PDI = 0.395 ± 0.026; Zeta potential = -27.8 ± 0.94 mV	*p-*value > 0.05; the difference is not significant

**Table 6:** Results of parameters obtained after fitting data release of QTF-loaded SEDDS to different kinetic models

Kinetic model	R^2^_adj_	AIC	Other parameters	Results
**Zero-order**	-21.8729	55.9229	k	2.263
**First-order**	0.9850	10.6613	k	0.151
**Higuchi**	-5.3309	48.0458	k	15.806
**Krosmeyer-peppas**	0.7160	30.3263	k	62.469
			n	0.124
**Weibull**	0.9940	7.2557	T	-8.582
			β	1.41
			T_d_	6.799
**Hopfenberg**	0.9862	10.3832	k	0.011
			n	1873.824

^*^R^2^_adj _indicated Adjusted coefficient of determination; AIC: Akaike information criteria; k: release rate constant; n: has a value of 1, 2, and 3 for a slab,

cylinder, and sphere, respectively; T: time; T_d_: the time required to dissolve 63,2% of the drug; and β: shape parameter.

## Conclusion

In this work, we developed a new self-emulsifying drug delivery system for the oral delivery of QTF. The use of D-optimal mixture design allowed to optimize the formulation with a minimal number of experiments. The obtained optimal formulation showed good physicochemical characteristics and good stability. The use of SEDDS as a drug delivery system has contributed to the improvement of the *in-vitro *dissolution and the intestinal absorption of QTF. Mathematical modeling of drug release profiles and TEM images have shown that the drug was released from oily droplets by diffusion and erosion mechanisms following the Weibull and Hopfenberg Models. These results indicate the suitability of the use of SEDDS as a delivery system for QTF. Additional studies are needed to confirm the role of this formulation in the improvement of the oral bioavailability of the drug.

## Author contributions

O.B.H.A., M.A.L, B.B., and S.S. conceived and designed the experiment. O.B.H.A. performed experimental work. O.B.H.A and M.A.L. Analyzed the experimental results. O.B.H.A and M.A.L. wrote the paper. All authors reviewed the paper.
